# Risky sexual behavior and self-rated mental health among young adults in Skåne, Sweden – a cross-sectional study

**DOI:** 10.1186/s12889-022-14823-0

**Published:** 2023-01-03

**Authors:** Anna Karle, Anette Agardh, Markus Larsson, Malachi Ochieng Arunda

**Affiliations:** Social Medicine and Global Health, Department of Clinical Sciences, Lund University, Jan Waldenströms gata 35, 205 02 Malmö, Sweden

**Keywords:** Young adults, Mental health, Depression, Anxiety, Sexual behavior

## Abstract

**Background:**

Risky sexual behavior is a public health challenge that significantly affects young people’s health and well-being in Sweden and throughout the world. Moreover, poor mental health, anxiety and depression among adolescents and young adults have increased in recent years. However, although hypothesized, the associations between general mental health and risky sexual behavior among young adults are less established. Thus, this study aimed to examine the association between self-rated mental health and risky sexual behavior among young adults in southern Sweden.

**Methods:**

Population-based, cross-sectional survey data from 2968 participants aged 18–30 years old residing in southern Sweden was used (response rate 42%). The survey included questions on sexual behavior, alcohol habits, sociodemographic background, and mental health. Logistic regression was used to examine the associations between mental health, depression, anxiety, and risky sexual behavior, stratified by sex (gender). Indicators for risky sexual behavior included not using a condom, non-condom use with casual partner, and multiple (≥2) sexual partners during the last year.

**Results:**

Generally, male participants rated their depression and anxiety levels considerably lower than their female counterparts. Poor mental health, high depression, and high anxiety scores (levels) were significantly associated with having multiple sexual partners among among female participants; adjusted odds ratios (aOR) was 1.3 (95% CI 1.01 to 1.71). However, findings among males were not statistically significant. Furthermore, overall results indicated that higher depression and anxiety scores were associated with 1.4 and 1.6 higher odds, respectively, of not using condom with a casual partner in the most recent sexual encounter. Similarly, higher anxiety scores were associated with non-condom use in the latest sexual encounter, aOR 1.4 (1.1–1.7), but no significant gender-specific associations were found.

**Conclusion:**

The associations found between poor mental health factors and multiple sex partners among females warrant consideration in future public health interventions. Further research to increase the understanding of the causal mechanisms that link mental health factors and risky sexual behavior, especially multiple sex partners, among young adult females is needed to support evidence-based interventions.

## Introduction

Risky sexual behavior is a global public health challenge that contributes substantially to the increased risk of sexually transmitted infections (STI) and/or unwanted pregnancies [1, 2]. In 2020, the World Health Organization (WHO) estimated that there were over 370 million new infections of STIs, such as chlamydia, gonorrhoea, and trichomoniasis, and that over 37 million lived with HIV globally [1, 3]. Young adults and adolescents are considered to constitute some of the most vulnerable groups [4]. Most of the STI infections are a consequence of risky sexual behaviors, which in turn have been hypothesized to be associated with factors such as alcohol consumption, substance use, and poor mental health – especially depression and psychological distress [5, 6]. Risky sexual behavior has far-reaching health and reproductive consequences that include cervical cancer, stillbirths, and maternal and neonatal deaths, all of which are major global health burdens [7, 8].

As with all human behavior, sexual behavior is complex and requires an in-depth understanding to guide interventions and policy making to reduce the spread of STIs and the prevalence of unwanted pregnancies. Like many countries, Sweden has a national strategy to minimize the spread and effect of HIV and other STIs, and in the strategy young adults are a major target group for prevention interventions [9, 10]. Studies have shown that young people across the globe engage in unprotected sex, sex with casual partners and multiple sexual partners are risky sexual behaviors [11–14]. A larger national survey in Sweden showed that only half of the young people had used a condom during their last sexual encounter with a casual partner [15], despite the fact that the majority of Swedish youth receive compulsory sexual education in school. In Sweden, the highest prevalence of STIs is found among adolescents and the younger adult population. Gonorrhea has increased by about 15% per year since 2015 with the greatest increase among those under 30 years of age [16]. Further, 80% of all chlamydia cases in Sweden are found among individuals aged 15–29 years [17].

Studies from across the world have found associations between depressive symptoms and risky sexual behavior, such as inconsistent condom use (occasional use or non-use) multiple sexual partners, and HIV infection among youths [12–14, 18, 19]. However, a meta-analysis of 34 studies in 2001 found limited evidence that poor mental health was associated with increased risky sexual behavior, and the review recommended further research after failing to dismiss potential associations [20]. A more recent study in the US found a negative association between casual sex and psychological well-being [21]. Furthermore, a 2006 study in the US found more depressive symptoms among females engaged in casual sex than among their male counterparts [22]. Addressing mental health issues is critical for safer sexual behavior [23]. In Sweden, there has been a steady decrease in self-reported mental well-being among young people 16–25 years old since 2015, especially among young women [24]. According to the Association of Sexuality Education in Sweden (RFSU), young adults aged 18–29 constitute the age group with the highest frequency of risky sexual behavior, and the highest prevalence of STIs and unwanted pregnancies [25]. This age group represents a critical development phase, both socially and psychologically, with opportunities to take control over one’s own life and make important decisions regarding education, career and relationships, as well as test boundaries and to mature.

Various hypotheses have been suggested to explain a potential association between poor mental health and risky sexual behavior. For this study, we adopted Kotchick’s and Shaffer’s framework (2001) that argues that risky sexual behavior among young people is primarily influenced by three interrelated systems; the self, the familial, and the extra-familial systems [26]. The authors posit that the self-system is composed of individual biological, psychological and behavioral factors that directly or indirectly shape one’s behavior [26]. Relevant biological factors would include age and sex. Behavioral factors, such as alcohol use has been reported to have complex relationship with risky sexual behaviors [27, 28]. In 2018, 22% of 16–29-year-olds were considered at-risk consumers of alcohol in Sweden [29]. In another larger study, 19% of young people reported having consumed alcohol in connection with their latest sexual encounter [15]. The familial system emphasizes family factors, such as socioeconomic factors, e.g., the level of education, background factors, e.g., parental place of birth, and family structure, e.g., whether a person grew up with one or both parents. The extra-familial system includes the social environment outside the family, such as peers, school and community [26]. This current study adapted Kotchick’s and Shaffer’s theoretical framework in the selection of variables used for analysis [26].

This study aimed to investigate the associations between poor mental health and risky sexual behavior among young adults, aged 18–30 years in southern Sweden. More specifically, the study sought to examine the associations between self-rated mental health and three sexual behaviors defined as risky, namely, non-condom use on latest (most recent) sexual encounter, non-condom use on latest sexual encounter with a casual partner, and multiple sexual partners during the last 12 months. We also included other factors, such as sociodemographic factors and alcohol consumption as potential confounders in the study since they can influence sexual behavior.

## Methods and materials

### Study design

This cross-sectional study was part of a larger population-based study on lifestyle, sexual behavior, and health among young adults in southern Sweden.

### Setting and participants

The survey was conducted in Skåne, the southernmost region of Sweden among individuals aged 18–30 years old as of March 2013. In 2013, at the time of data collection, the population of Skåne accounted for about 13% of the total registered population in Sweden and there were approximately 206,000 individuals in Skåne between the ages of 18 and 29 [30].

### Data collection

The data was collected by sending a survey invitation letter via post to 7000 potential individual participants aged 18–30 years as of March 2013. The participants were randomly selected from the National Swedish Population Register (Svensk Folkbokföring). The letter introduced the survey and clarified that participation was voluntary and that anonymity of participants was guaranteed throughout the study process. Contact information to the responsible researcher, as well as to a contact person, a midwife, whom participants could consult about the subjects brought up in the questionnaire, was also enclosed. The letter contained a link to a server that participants could use to answer the questionnaire online. Three reminders to complete the survey were sent to participants, one of which contained a printed version of the survey. The possibility to return the printed version was given. Further details about the study population can be obtained from a previous sub-study by Sundbeck and colleagues in 2016 [31], using data from the same survey. A total of 2968 participants responded to the survey, representing about 42% response rate. The questionnaire contained a total of 79 questions, covering the following subjects: sociodemographic background, social capital, violence, use of alcohol and drugs, sexual harassment and abuse, sex and sexuality, travelling, sensation seeking, and general and mental health. Data was collected between January and March 2013.

### Variables

The selection of factors examined in this study was guided by Kotchick’s and Shaffer’s [26] framework and the availability of variables in the dataset. For example, the framework postulates that self-system factors include individual biological, psychological and behavioral factors, which in this study were represented by the sex and age of the participants. Self-rated mental health and alcohol consumption were included as psychological and behavioral factors. Similarly, the familial system included parental education, and family structure, among others, while the extra-familial systems included participants’ education and birthplace along with other factors detailed below.

#### Dependent variables – risky sexual behavior

##### Non-condom use on latest (most recent) sexual encounter in Sweden

The questionnaire contained several questions on sexual behavior. This variable was derived from the question *“Did you or your partner use condom to protect you from sexual transmitted diseases during your latest (most recent) sexual encounter in Sweden?*”. Condom use on latest sexual encounter in Sweden was categorized as ‘yes’ or ‘no’.

##### Non-condom use with casual partner on latest (most recent) sexual encounter in Sweden

This variable was created from two questions, use of condom above and the question “*Which relationship did you have with the person you had latest sex within Sweden?”*. Casual sexual partner was defined as anyone with whom the participants had sex but with whom the participant was not in a formal relationship. Participants who used a condom during their latest casual sex were categorized as ‘yes’ and those who did not were categorized as ‘no’.

##### Multiple sexual partners

This variable was generated from the question *“How many sexual partners have you had in the last 12 months in Sweden?”* The number of sexual partners that the participants had had during the last 12 months was dichotomized into ‘0-1 sexual partners’ and ‘≥2 sexual partners (multiple sexual partners)’. Although studies indicate inconsistent findings, Lundberg et al. (2011) found associations between psychological distress, depression, and multiple sex partners [19].

#### Independent variables – mental health

##### Total mental health score

Total mental health scores were calculated using the mean scores from 24 survey questions on mental health symptoms. The questions were derived from the Hopkins Symptoms Checklist (HSCL-25) [32], a self-administered questionnaire based on an outpatient psychiatric rating scale - SCL-90 [33]. 14 of the questions concerned depressive symptoms and 10 concerned anxiety symptoms. In each question, participants were asked to rate their response concerning symptoms experienced during the past 30 days, using a scale of 1 to 4, where 1 = “not at all” (meaning it was not a problem) and 4 = “extremely” (meaning it was a major problem). A high mean score therefore represented more symptoms (poor mental health), whereas a low mean score represented fewer symptoms (good mental health). The variable was dichotomized by categorizing the means of the participants’ total mental health scores as a ‘high score’ or ‘low score’, based on a median split of the distribution of the participants’ individual mean mental health scores. A similar methodology using a cut-off point to examine sub-group differences has been used in previous studies [31, 34], as the instruments do not designate any specific score that corresponds to poor mental health. The HSCL-25 and SCL-90 self-assessment instruments have been used extensively for mental health screenings globally for decades and have good reliability and high validity [32, 33, 35].

##### Depression score and anxiety score

14 of the 24 items about mental health were questions about depressive symptoms. The means from the participants’ responses to these questions were dichotomized in the same way as the ‘Total mental health score’ above. Those with mean scores above the median were placed in the ‘high depression score’ group, and those below in the ‘low depression score’ group. Similarly, 10 of the 24 items about mental health were questions about anxiety. The responses to these questions were dichotomized in the same way as the ‘Total mental health score’ above. Those with mean scores above the median were placed in the ‘high anxiety score’ group, and those below in the ‘low anxiety score’ group.

##### Heavy episodic alcohol consumption (heavy episodic drinking)

This variable was derived from the question *“How often do you consume at least five (if respondent is male) or four (if respondent is female) glasses during the same drinking occasion, during the last 12 months?”*. Six choices were provided, which included; “daily”, “every week”, “2-4 times a month”, “every month”, “less often than once a month” and “never”. The responses were dichotomized as ‘Once a month or more’ and ‘Less often than once a month or never’. Heavy episodic drinking referred to the frequency and quantity of alcohol consumed and was defined as five glasses for males and four glasses for females, per episode of alcohol drinking [36, 37]. Heavy episodic drinking has been reported to be a risk factor for both risky sexual behavior [27, 28] and poor mental health [38].

##### Alcohol use in conjunction with latest sexual encounter in Sweden

Alcohol use in conjunction with latest sexual encounter in Sweden was categorized as ‘Yes’ and ‘No’.

#### Background variables

##### Gender

Participants were asked to categorize their gender (sex) as male or female as designated at birth. Seven participants did not specify their gender and were excluded from the study.

##### Age

Participants aged 18–30 years as of March 2013 were included in the study. To examine the effect of age difference in our study, we dichotomized the participants’ age into two age groups based on the WHO definition of youth, and by further extension, “post youth”, such that ‘18–24′ represented youth and ‘25–30′ years old represented “post-youth” or young adults.

##### Participants´ birthplace

Participants’ place of birth was dichotomized as ‘Sweden’ if born in Sweden and ‘abroad’ if born abroad.

##### Parents’ place of birth

Parents’ place of birth had three response options in the questionnaire: ‘Both parents born in Sweden’; ‘One parent born in Sweden’; and ‘Both parents born in another country’. These responses were dichotomized into: ‘Both parents born in Sweden’ (reference category), and ‘At least one parent born abroad’. Studies have associated Swedish-born and foreign backgrounds with diverse sexual attitudes and behaviors, as well as differences in socioeconomic and community factors [39, 40].

##### Parents’ level of education

Parental levels of education were dichotomized into ‘High or middle school’ and ‘University or other higher education’. Level of education of parent can be a predictor factor for family socioeconomic background, child development and the individual’s educational attainment later in life [41].

##### Participants’ level of education

This variable was also dichotomized into ‘High or middle school’ and ‘University or other higher education’.

##### Adults present during childhood

Information on the adult(s) with whom the participants had spent most time with during childhood was also recorded. Response options included one parent, or both parents, and other persons. Responses were dichotomized into ‘Both parents’ and ‘One parent, or other person’. Studies have reported diverse effects of family structure in relation to sexual behavior [42].

### Data analysis

Analysis was conducted using SPSS 25.0 (IBM Corp. Armonk, NY: IBM Corp). Chi-square tests were used to examine group differences for the category variables. Independent Samples T-tests were used to compare the means of the mental health scores between males and females. Significance level of *p*-value < 0.05 was used. To correct biases in the survey sample and to take into account population differences, we weighted the data using variables gender (sex), age and place of birth. Using 2013 data for age-group 18–30 years in Skåne from the Swedish statistical authority (SCB), we weight data to adjust our sample to population characteristics to improve representativeness. Logistic regression analysis stratified by gender was used to calculate odds ratios (OR) at 95% confidence intervals (CI) for the association between independent (mental health variables and others) and dependent variables (risky sexual behaviors). The crude odds ratios obtained between the independent and dependent variables were then adjusted for all background variables in the study. Cases with missing values were excluded from the analyses. Assessment of the missing data indicated about 11% missing data across the three main dependent risky sexual behavior variable variables and about 1% missing across the mental health and background variables. Missing data was relatively randomly distributed and thus had minimal to no effect on the results.

## Results

Table 1 below shows the distribution of the background, lifestyle, and risky sexual behavior variables by the exposure variables, mental health, depression and anxiety, including *p*-values with 95% confidence intervals (total *N* = 2961). In this sample, about 60% of participants were aged 18–24 years old, and 40% were 25–30 years old and there were slightly more females (58.5%) than males. Nearly 86% of the study participants were born in Sweden and 72% of the parents were also born in Sweden. Regarding background variables, females and persons in the younger age-group 18–24 years more frequently reported poor mental health and high anxiety or depression scores, compared to males and persons in the older age-group (25–30 years), respectively, *(P < 0.05).* Similarly, we detected significant associations between participants’ level of education and adult present during childhood and certain mental health variables (see Table 1).

**Table 1:** Distribution of background factors, risky sexual behavior, and lifestyle factors among young adults in Sweden by self-reported mental health status (unweighted), 95% confidence interval.

Variables	Subtotals, (%)	Mental health			Depression			Anxiety		
		Low (good), n (%)	High (poor), n (%)	*X*^*2*^	Low, n (%)	High, n (%)	*X*^*2*^	Low, n (%)	High, n (%)	*X*^*2*^
***Gender***										
Male	41.5	789 (52.0)	430 (30.1)	0.001	707 (42.5)	511 (40.0)	0.182	898 (48.8)	321 (29.1)	0.001
vFemale	58.5	728 (48.0)	998 (69.9)		958 (57.5)	766 (60.0)		943 (51.2)	783 (70.9)	
*Missing, n*	*7*									
***Age (****years****)***										
25–30	39.5	634 (42.2)	518 (36.6)	0.002	687 (41.7)	463 (36.5)	0.004	744 (40.8)	408 (37.3)	0.061
18–24	*60.5*	869 (57.8)	899 (63.4)		960 (58.3)	806 (63.5)		1081 (59.2)	687 (62.7)	
*Missing, n*	*29*									
***Participants’ birth place***										
Sweden	86.5	1313 (86.9)	1226 (86.5)	0.765	1433 (86.5)	1103 (87.1)	0.650	1603 (87.4)	936 (85.6)	0.154
Abroad	13.5	198 (13.1)	191 (13.5)		224 (13.5)	164 (12.9)		231 (12.6)	158 (14.4)	
*Missing*	*24*									
***Parents’ birthplace***										
Both born in Sweden	72.0	1102 (72.9)	1012 (71.3)	0.345	1206 (72.7)	905 (71.3)	0.395	1353 (73.8)	761 (69.4)	0.010
One or both born abroad	28.0	410 (27.1)	407 (28.7)		452 (27.3)	364 (28.7)		481 (26.2)	336 (30.6)	
*Missing, n*	*21*									
***Participants’ educational level***										
University/other higher ed.	44.9	702 (46.4)	615 (43.3)	0.090	778 (46.9)	536 (42.1)	0.011	852 (46.4)	465 (42.3)	0.029
High school or lower	55.1	812 (53.6)	807 (56.8)		882 (53.1)	736 (57.9)		984 (53.6)	635 (57.7)	
*Missing, n*	*17*									
***Parents’ educational level***										
University/other higher	60.1	887 (58.9)	871 (61.6)	0.125	972 (58.9)	783 (61.9)	0.098	1102 (60.3)	656 (60.0)	0.873
High school or lower	39.9	620 (41.1)	542 (38.4)		679 (41.1)	482 (38.1)		725 (39.7)	437 (40.0)	
*Missing*,* n*	*34*									
	χ^2^ – *p*-value from chi-square test, Low - means *good* mental health variable, High – means *poor*									
***Adults present during childhood***										
Both parents	77.0	1231 (81.4)	1036 (72.5)	0.001	1334 (80.4)	927 (72.8)	0.001	1473 (80.3)	791 (71.8)	0.001
One parent or other persons	23.0	281 (18.6)	391 (27.5)		325 (19.6)	346 (27.2)		362 (19.7)	310 (28.2)	
*Missing, n*	*16*									
***Heavy episodic drinking***										
Once a month or more	51.1	698 (51.2)	659 (51.0)	0.931	795 (52.9)	560 (48.7)	0.032	867 (52.1)	490 (49.4)	0.177
Less than once a month	48.9	666 (48.8)	633 (49.0)		708 (47.1)	590 (51.3)		797 (47.9)	502 (50.6)	
*Missing, n*		*307*								
***Alcohol use in conjunction with most recent sexual encounter in Sweden***										
Yes	20.1	256 (18.7)	275 (21.8)	0.044	282 (18.7)	247 (2.0)	0.039	308 (18.7)	223 (22.7)	0.014
No	79.9	1116 (81.3)	986 (78.2)		1224 (81.3)	876 (78.0)		1341 (81.3)	761 (77.3)	
*Missing, n*	*324*									
***Condom use during most recent sexual occasion in Sweden***										
Yes	23.6	324 (24.0)	294 (23.4)	0.755	344 (23.1)	272 (24.4)	0.454	407 (25.0)	211 (21.6)	0.052
No	76.4	1028 (76.0)	960 (76.6)		1143 (76.9)	843 (75.6)		1223 (75.0)	765 (78.4)	
*Missing, n*	*354*									
***Condom use with casual partner during most recent sexual encounter in Sweden (n = 803)***										
Yes	40.8	170 (44.4)	155 (37.8)	0.060	177 (44.6)	148 (37.5)	0.042	213 (44.9)	112 (35.1)	0.006
No	59.2	213 (55.6)	255 (62.2)		220 (55.4)	247 (62.5)		261 (55.1)	207 (64.9)	
*Missing, n*	6									
***Number of sexual partners during last 12 months in Sweden***										
0–1	70.6	1008 (73.9)	847 (67.1)	0.001	1107 (73.9)	746 (66.3)	0.001	1203 (73.2)	652 (66.3)	0.001
≥ 2	29.4	356 (26.1)	416 (32.9)		391 (26.1)	379 (33.7)		440 (26.8)	332 (33.7)	
*Missing, n*	*333*									
	χ^2^ – *p*-value from chi-square test (95% CI), For mental health variables, low - means *good*, high – means *poor*									

Lifestyle factors, such as heavy episodic drinking and use of alcohol during the most recent sexual encounter also indicated significant associations with at least one mental health variable *(P < 0.05).* About half (51%) of the participants had at least one episode of heavy drinking every month, while only 20% of the participants had sexual encounter while under the influence of alcohol. Significant associations were also found between mental health variables and outcome variables (risky sexual behaviors), particularly multiple sexual partners and non-condom use during most recent sexual encounter with a casual partner.

Table 2 shows that there was a statistically significant difference between how males and females rated their mental health. The mean score for males was significantly lower compared to females across all three mental health variables, including depression and anxiety.

**Table 2:** Comparing means of self-rated mental health scores of participants, by gender

	Total			Male			Female			Ind. T-test
	n	Mean	sd	n	Mean	sd	n	Mean	sd	*p-value*
*Total mental health*	2949	1.77	0.55	1219	1.60	0.47	1726	1.89	0.57	< 0.001
Missing	19			9			7			
*Depression*	2946	1.83	0.63	1218	1.63	0.55	1724	1.98	0.64	< 0.001
Missing	22			10			9			
*Anxiety*	2949	1.71	0.50	1219	1.58	0.42	1726	1.82	0.53	< 0.001
Missing	19			9			7			

n - number, sd - standard deviation, Ind. T-test – Independent Samples T-test, level of significance < 0,05

Table 3 presents crude odds ratios (OR) with 95% confidence intervals (CI) for the relationship between background, lifestyle and mental health variables and the risky sexual behavior variables stratified by gender. Among females in this study, poor mental health and high depression and anxiety scores showed significant associations with having multiple partners, with crude odds ratios ranging between 1.4–1.5.

**Table 3:** Crude odds ratios (cOR), and 95% confidence intervals (CI), for associations between sociodemographic, lifestyle, and mental ill health symptom scores, and the different risky sexual behaviors in Swedish young adults, stratified by gender. Risky sexual behaviors

	No condom on latest sexual encounter. cOR (95% CI)		No condom use with casual partner on latest sexual encounter. cOR (95% CI)		Multiple sexual partners. cOR(95% CI)	
	Male	Female	Male	Female	Male	Female
***Age (****years)*						
18–24	ref	ref	ref	ref	ref	ref
25–30	**1.5 (1.1–2.0)**	1.3 (1.0–1.7)	1.2 (0.8–1.9)	1.4 (0.8–2.2)	**0.5 (0.4–0.7)**	**0.5 (0.4–0.7)**
***Parents’ place of birth***						
Both born in Sweden	ref	ref	ref	ref	ref	ref
One or both born abroad	**0.4 (0.3–0.5)**	**0.6 (0.5–0.9)**	**0.4 (0.2–0.6)**	0.6 (0.4–1.0)	1.0 (0.7–1.4)	0.8 (0.6–1.1)
***Participants’ educational level***						
University or other higher	ref	ref	ref	ref	ref	ref
High school or lower	0.8 (0.6–1.1)	1.0 (0.8–1.3)	0.6 (0.4–1.0)	1.4 (0.9–2.1)	1.1 (0.8–1.4)	**1.5 (1.2–1.9)**
***Parents’ educational level***						
University/other higher	ref	ref	ref	ref	ref	ref
High school or lower	1.0 (0.8–1.3)	1.1 (0.8–1.4)	1.2 (0.8–1.8)	1.4 (0.9–2.3)	**0.7 (0.5–0.9)**	1.1 (0.8–1.2)
***Adults present during childhood***						
Both parents	ref	ref	ref	ref	ref	ref
One parent or other person	0.9 (0.7–1.3)	1.2 (0.9–1.6)	0.8 (0.5–1.2)	1.4 (0.8–2.3)	**2.1 (1.5–2.8)**	**1.4 (1.1–1.8)**
***Heavy episodic drinking***						
Less than once a month	ref	ref	ref	ref	ref	ref
Once a month or more	**1.4 (1.01–1.81**)	1.0 (0.8–1.3)	1.4 (0.9–2.1)	1.0 (0.7–1.6)	**2.0 (1.5–2.6)**	**2.0 (1.6–2.5)**
***Alcohol use in conjunction with last sexual occasion in Sweden***						
No	ref	ref	ref	ref	ref	ref
Yes	**0.7 (0.5–0.9)**	**0.5 (0.4–0.7)**	0.8 (0.5–1.2)	1.2 (0.8–1.8)	**3.2 (2.4–4.3)**	**3.2 (2.4–4.2)**
***Mental ill health symptom scores***						
Low (good health)	ref	ref	ref	ref	ref	ref
High (poor health)	0.9 (0.7–1.2)	0.9 (0.7–1.2)	1.4 (0.9–2.1)	1.0 (0.6–1.5)	**1.3 (1.01–1.80)**	**1.5 (1.2–1.9)**
***Depression score***						
Low (good health)	ref	ref	ref	ref	ref	ref
High (poor health)	0.9 (0.7–1.2)	0.9 (0.7–1.2)	1.4 (0.9–2.1**)**	1.3 (0.9–2.0)	**1.4 (1.1–1.8)**	**1.5 (1.2–1.8)**
***Anxiety score***						
Low (good health)	ref	ref	ref	ref	ref	ref
High (poor health)	1.2 (0.9–1.7)	1.1 (0.8–1.4)	**1.6 (1.02–2.52)**	1.3 (0.9–2.0)	1.3 (1.0–1.8)	**1.4 (1.2–1.8)**

*OR Odds ratio, *CI* Confidence interval, bold means statistically significant observation

Furthermore, significantly increased odds of not using a condom were seen in the older (25–30 years) age group for males. Having both parents born abroad was associated with 40–60% (unadjusted) likelihood of using condom in latest sexual encounters for both males and females, hence protective. Furthermore, among both males and females, heavy episodic drinking and alcohol use in conjunction with last sexual occasion in Sweden were significantly associated with having multiple sexual partners. Being in the older age group decreased the odds of having multiple sex partners in the last 12 months for both genders.

In Table 4 below, after adjusting for all background and lifestyle factors, high depression and anxiety symptoms showed significant associations with having multiple sex partners; in the overall population, with the aOR ranging from 1.2 to 1.3 (95% CI 1.01 to 1.50). Gender specific findings for the association between poor mental health, high depression, high anxiety and multiple sex partners were significant only among females, (aOR 1.3 (95% CI 1.01 to 1.71). Corresponding associations among males were not statistically significant for all mental health variables, aOR 1.1 to 1.3 (95% CI 0.8 to 1.8). Generally, having high anxiety was significantly associated with; non-condom use on the latest (most recent) sexual encounter, non-condom use with casual partner on most recent sexual encounter; overall aOR for these associations ranged from 1.3 to1.6 (95% CI 1.03 to 2.23). Overall, high depression was also significantly associated with not using a condom in the latest sexual encounter with casual partner (aOR 1.4 (95% CI 1.04–1.96).

**Table 4:** Association (adjusted odds ratio (aOR) and 95% confidence interval (CI)) between mental health factors and sexual risk behaviors, adjusted for lifestyle and sociodemographic factors in Swedish young adults, stratified by gender

	No condom on latest sexual encounter (aOR, 95% CI)			No condom on latest sexual encounter with casual partner (aOR, 95% CI)			Multiple sexual partners (aOR, 95% CI)		
	Overall	Male	Female	Overall	Male	Female	Overall	Male	Female
High mental health score (poor health)	1.1 (0.9–1.3)	0.9 (0.7–1.3)	1.0 (0.8–1.3)	1.4 (1.0–1.9)	1.4 (0.9–2.2)	1.1 (0.7–1.7)	1.2 (1.0–1.5)	1.1 (0.8–1.5)	**1.3 (1.01–1.67)**
High depression score (poor health)	1.0 (0.7–1.3)	1.0 (0.7–1.3)	1.0 (0.8–1.4)	**1.4 (1.04–1.96)**	1.4 (0.9–2.2)	1.4 (0.9–2.2)	**1.2 (1.01–1.50)**	1.1 (0.8–1.5)	**1.3 (1.03–1.71)**
High anxiety score (poor health)	**1.4 (1.1–1.7)**	1.3 (0.9–1.9)	1.1 (0.9–1.5)	**1.6 (1.2–2.23)**	1.4 (0.9–2.5)	1.4 (0.9–2.2)	**1.3 (1.03–1.50)**	1.3 (0.9–1.8)	**1.3 (1.04–1.64)**

Adjusted for age, parental place of birth, adults lived with while growing up, participants and parental level of education, heavy episodic drinking, and alcohol use in conjunction with last sexual encounter

Bold represent statistically significant results

## Discussion

This study aimed to examine the associations between poor mental health (exposure) and risky sexual behaviors (outcome) among young adults aged 18–30 years in Skane region, Sweden. Overall, the findings indicated a significant 20–30% increase in the odds of having multiple sex partners in the last 12 months among young adults who had high depression and anxiety scores, compared to those with low depression and anxiety scores. When stratified by gender, the above findings concerning multiple sex partners were statistically significant for all poor mental health variables among females only, with adjusted odds ratios 1.3 times higher compared to females with lower mental health, depression, and anxiety scores. The overall results also indicated significant associations between high self-reported anxiety and not using a condom on latest sexual encounter, including during latest sex with casual partner.

To our knowledge, this is one of the first studies in Sweden to examine the effect of mental health on risky sexual behavior among young adults aged 18–30 years. Studies conducted elsewhere that have investigated the associations between self-reported depressive, anxiety or poor mental health symptoms and having multiple sex partners or non-use of condom during sexual encounters among adolescents, young adults, and adults largely concur with our findings [13, 14, 43–45].

In agreement with our findings, a recent study among predominantly young adults across England by Coyle et al. found 1.7 higher odds of condomless sex with multiple sex partners among females with anxiety symptoms [43]. Another study in Finland, a similar setting as Sweden, also found associations between depression and multiple sex partners and non-use of contraceptives, including condoms, among mid-teens [44]. However, Ramrakha and colleagues in New Zealand examined the associations between having multiple sex partners and later (after 12 months period) mental disorders (depression and anxiety) [45]. Before adjusting for prior mental disorders, they found a strong association (twice higher odds) between having > 2.5 sexual partners and anxiety only [45]. However, this association disappeared after adjusting for prior mental disorder. Although the outcome variables (mental disorders) in the Ramrakha et al. study were the predictor in our study, the significant results before controlling for the effect of prior mental health disorders indicate the need for further research [45]. Given our findings and the studies referred to above, it would be plausible to suggest that having multiple sexual partners is a more robust indicator for risky sexual behavior - perhaps partly because it is easier to measure and recall.

In line with our findings, it is plausible that depression and anxiety are associated with risky sexual behaviors, either by driving these behaviors via feelings of worthlessness and hopelessness associated with depression, or through blurred judgement impeding the evaluation of the probable risks [43, 46, 47]. The idea that sexual relationships can alleviate the burden of emotional distress has been discussed by Ein-Dor et al. (2012) [46]. Further, self-medication with alcohol is a common problem in mood and anxiety disorders [48]. On the other hand, other studies have argued that risky sexual behaviors drive the depressive and other poor mental health symptoms [21, 45, 49, 50]. Notably, all these studies utilized different standard mental health screening tools. However, because of the cross-sectional nature of the studies, the direction of causality cannot be established, and it is thus also plausible that causality goes in either direction.

We also found associations between anxiety and depression and non-use of condom during casual sex, and this is consistent with a case-control study conducted in Brazil, which found risky sexual behavior, such as unprotected sex, to be associated with anxiety and depression among adult men 18–66 years old [51]. It is also possible that certain risky sexual behaviors, such as condomless casual sex, could be acts of self-harm. Studies in Taiwan and Sweden have indicated that risky sexual behaviors can be self-destructive acts [52, 53]. Nonetheless, further studies are necessary to ascertain such hypotheses. Non-use of condom could also be due to a perception that HIV prevalence in Sweden is low, while the more commonly existing STIs are curable [54, 55]. Condom use is a complex factor that varies among individuals over time, situations and partners, and more in-depth questions about condom use in the survey may have yielded other results. As suggested by Kalichman and Weinhardt, the association between poor mental health and risky sexual behavior may be more easily observed at an event level, thus requiring event-level analysis [56].

Using a clinical diagnosis would have improved the validity of the findings but that would have required face-to-face clinical interview by a health professional. The HSCL-25 and SCL-90 items used in this study are tools used for self-rated mental health, and the scales do not yield clinical diagnoses. Therefore, it is unknown how many participants with high scores on poor mental health would meet clinical diagnostic criteria for anxiety or depression. The findings nonetheless show that high scores on self-rated mental health have implications for risky sexual behavior. The gender differences in self-rated mental health shown in this study confirm previous findings from multiple epidemiological studies indicating females are more likely to develop poor mental health than males [57, 58].

As theorised by Kotchick [26], a myriad of factors interacting in a system affect young people’s risky sexual behavior. In this study several background factors indicated significant associations with risky sexual behavior. For instance, belonging to the older age-group (25–30 years) was associated with higher odds of unprotected sex and decreased odds of multiple sex partners in the unadjusted results. This could be explained by the increased likelihood of the participants in the older group being in a monogamous relationship [59]. Another interesting observation was that having one or both parents born abroad decreased the odds of unprotected sex during latest sexual encounter. A possible explanation could be that parents from other countries where STI/HIV may be more prevalent than in Sweden would promote condom-use and/or abstinence to their children. HIV awareness has generally been low in Sweden [55, 60]. Although inconsistent in the literature, the strong association between drinking alcohol and having multiple sexual partners found in this study agrees with findings by Cooper (2002) [28].

Although our study did not investigate the mechanism through which poor mental health symptoms elicit or augment risky sexual behavior, mental health factors could potentially be both a risk factor for, and an outcome of, risky sexual behavior. Future studies using longitudinal designs could provide further insight into the causality between these factors and the degree to which they influence each other. Additionally, qualitative studies and study designs that allow event-level analysis would also be useful in the quest to further understand complex human behaviors, such as condom use. More comprehensive interventions aimed at improving mental health in Sweden may be needed to reduce risky sexual behavior and spread of STIs and HIV. In the last 7 years prior to 2021 in Sweden, poor mental health has increased by over 10%, indicating inadequacies of current interventions [61].

### Strengths and limitations

The study was population-based, involved random selection of participants, and had a relatively large sample size with sufficient power, factors which enhance the study’s generalizability. However, the participation rate (42%) was relatively low, and the findings should be interpreted with caution. Nonetheless, the participation rate was similar to the rates currently obtained by epidemiological surveys [62, 63]. A meta-analysis study of over 1000 online surveys found an average participation rate of 44% [63], and this needs to be explored further not only identify the reasons for low response rates but also to identify evidence-based solutions to improve online survey participation. Possible reasons for non-response in the current study could be lack of sufficient time to respond to a long questionnaire, unwillingness to disclose sensitive information and inadequate Swedish language skills, among others. Possible solutions to improve response rates could include using shorter surveys of about 10 minutes, semi-automatic log-in and incentives as highlighted in a recent review by Sammut and colleagues [64]. However, given the survey tools used in this study, the random selection of participants and the broad definition of the study outcome, non-response is likely to be random across all groups. Additionally, the use of standard instruments for assessing mental health improves the validity and generalizability of the study. We also applied weights to correct for probable biases in the survey sample and to take into account population differences using key identified variables and these included age, gender and place of birth.

A potential limitation in the survey methodology is the possibility of recall bias. However, limiting the mental health experiences to the last 30 days and focusing on sexual risky behaviors that are not easily forgotten and that occurred during the last 12 months likely minimized any recall bias. The fact that condom use or non-use was based on latest sexual encounter may have further decreased recall bias. However, a one-time sexual encounter does not necessarily indicate the full picture of the participants’ condom-using habits [65], and thus the findings should be interpreted cautiously.

Most participants in the study had Swedish-born parents, parents with higher education, and had grown up with both parents. Generally, this participation pattern reflects the demographic characteristics of the study population and is consistent with that of other epidemiological studies [66]. However, there is a possibility of selection bias against less educated and foreign-born inidviduals, and this participation pattern should be kept in mind when drawing conclusions based on our results. Further, the proportion of female participants was greater than males (58.5% vs. 41.5%). Nonetheless, the gender pattern consisting of more female participants compared to males is a common challenge of many web-based epidemiological surveys [63]. Future surveys could be designed to overcome such methodological challenges. Furthermore, our weighted results showed negligible differences from the unweighted results. As having multiple sexual partners is generally regarded as socially unacceptable behavior, this could have elicited socially desirable responses to the survey question, thus biasing our findings. However, the anonymous nature of the survey could have minimized such biases.

## Conclusion

Good mental and sexual health are critical for the development and well-being of young people. Mental health care and efforts for prevention of unwanted pregnancies and STIs/HIV could consider new strategies to further reduce poor mental health and its associated tendencies for risky sexual behaviour. Further research to increase the understanding of the causal mechanisms linking mental health factors and sexual risky behaviors is needed to enhance evidence-based interventions especially among females.

## Data Availability

Data can be accessed by contacting Prof. Anette Agardh via anette.agardh@med.lu.se.

## Abbreviations

WHO: World Health Organisation
STI: Sexually transmitted infections
HIV: human immunodeficiency virus
HSCL: Hopkins Sytom Checklist
SDG: Sustainable development goals

## References

[1] World health organization. Sexually transmitted infections (STIs). Key facts. Available from: https://www.who.int/news-room/fact-sheets/detail/sexually-transmitted-infections-(stis) . Cited 9 April 2022.

[2] World Health Organization. Sexual health and its linkages to reproductive heath: an operational approach. Geneva. 2017; Available from: https://apps.who.int/iris/handle/10665/258738.

[3] UNAIDS (2021). Global HIV statistics.

[4] World health organization. Global health sector strategy on sexually transmitted infections 2016–2021. Towards ending STIs. Geneva; 2016. Available from: https://www.paho.org/en/documents/global-health-sector-strategy-sexually-transmitted-infections-2016-2021-towards-ending. Cited 9 April 2022

[5] Pittman D, Rush C, Litt S, Minges M, Quayson A. Psychological distress as a primer for sexual risk taking among emerging adults. Int J Sexual Health. 33(3):371–84. Available from. 10.1080/19317611.2021.1919950. 10.1080/19317611.2021.1919950 PMC10903698 38595742

[6] Foley JD, Vanable PA, Brown LK, Carey MP, DiClemente RJ, Romer D (2019). Depressive symptoms as a longitudinal predictor of sexual risk behaviors among African-American adolescents. Health Psychol.

[7] World Health Organization (2019). Adolescent pregnancy.

[8] Shepherd J, Peersman G, Weston R, Napuli I (2000). Cervical cancer and sexual lifestyle: a systematic review of health education interventions targeted at women. Health Educ Res.

[9] United Nations. Sustainable Development Goals - Goal 3: Ensure healthy lives and promote well-being for all at all ages. Available from: https://www.un.org/sustainabledevelopment/health/. Cited 10 May, 2022.

[10] Swedish Department of Social Affairs (2017). National Strategy againts HIV/AIDS and certain other cummunicable diseases.

[11] Steinberg L (2004). Risk taking in adolescence: what changes, and why? Adolescent brain development: vulnerabilities and opportunities. Annals of the New York Academy of Sciences.

[12] Lehrer JA, Shrier LA, Gortmaker S, Buka S (2006). Depressive symptoms as a longitudinal predictor of sexual risk behaviors among US middle and high school students. Pediatrics.

[13] Shrier LA, Harris SK, Sternberg M, Beardslee WR (2001). Associations of depression, self-esteem, and substance use with sexual risk among adolescents. Prev Med.

[14] Ramrakha S, Caspi A, Dickson N, Moffitt TE, Paul C (2000). Psychiatric disorders and risky sexual behaviour in young adulthood: cross sectional study in birth cohort. BMJ.

[15] Public Health Agency of Sweden. Sexuality and health among young people in Sweden. Solna; 2017. Contract No.: 02930–2017. Available from: https://www.folkhalsomyndigheten.se/publicerat-material/publikationsarkiv/s/sexuality-and-health-among-young-people-in-sweden/ . Cited 30 May, 2022

[16] Public Health Agency of Sweden (2017). Gonorrhea Infections 2017.

[17] Public Health Agency of Sweden (2017). Chlamydia Infections.

[18] Cooke T, Bastien G, Xu J, Owen J, Cunningham K, Rust G. Major depressive disorder and condom use in young adult females. HSOA J Psychiatry Depress Anxiety. 2016:2 Available from: https://pubmed.ncbi.nlm.nih.gov/30556061/. 10.24966/PDA-0150/100006 PMC6294131 30556061

[19] Lundberg P, Rukundo G, Ashaba S, Thorson A, Allebeck P, Ostergren PO (2011). Poor mental health and sexual risk behaviours in Uganda: a cross-sectional population-based study. BMC Public Health.

[20] Crepaz N, Marks G (2001). Are negative affective states associated with HIV sexual risk behaviors? A meta-analytic review. Health Psychol.

[21] Bersamin MM, Zamboanga BL, Schwartz SJ, Donnellan MB, Hudson M, Weisskirch RS (2014). Risky business: is there an association between casual sex and mental health among emerging adults?. J Sex Res.

[22] Welsh DP, Grello CM, Harper MS (2006). No strings attached: the nature of casual sex in college students. J Sex Res.

[23] Patel V, Flisher AJ, Hetrick S, McGorry P (2007). Mental health of young people: a global public-health challenge. Lancet.

[24] Public Health Agency of Sweden (2019). Statistics over adults mental health.

[25] Riksförbundet För Sexuell Upplysning (RFSU). Sex och unga vuxna. Available from: https://www.rfsu.se/sex-och-relationer/for-dig-som-undrar/sex-genom-livet/sex-och-unga-vuxna/ 2022.

[26] Kotchick BASA, Forehand R (2001). Adolescent sexual risk behavior: a multi-system perspective. Clin Psychol Rev.

[27] Cook RL, Clark DB (2005). Is there an association between alcohol consumption and sexually transmitted diseases? A systematic review. Sex Transm Dis.

[28] Cooper ML (2002). Alcohol use and risky sexual behavior among college students and youth: evaluating the evidence. J Stud Alcohol Suppl.

[29] Public Health Agency of Sweden (2019). Risk Consumption of Alcohol.

[30] Statistics Sweden (SCB) (2022). Statistikdatabasen.

[31] Sundbeck MEA, Mannheimer L, Miorner H, Agardh A (2016). Sexual risk-taking during travel abroad - a cross-sectional survey among youth in Sweden. Travel Med Infect Dis.

[32] Derogatis LR, Lipman RS, Rickels K, Uhlenhuth EH, Covi L (1974). The Hopkins symptom checklist (HSCL): a self-report symptom inventory. Behav Sci.

[33] Socialstyrelsen. (2014). Socialstyrelsens granskning av SCL-90 - Symptoms Checklist.

[34] Sundbeck MAA, Ostergren PO (2017). Travel abroad increases sexual health risk-taking among Swedish youth: a population-based study using a case-crossover strategy. Glob Health Action.

[35] Derogatis LR, Lipman RS, Covi L. SCL-90: an outpatient psychiatric rating scale--preliminary report. Psychopharmacol Bull 1973;9(1):13–28. Available from: https://pubmed.ncbi.nlm.nih.gov/4682398/ . 4682398

[36] Läkemedelsverket. Läkemedelsboken: Swedish Medical Products Agency,; 2018. Available from: https://lakemedelsboken.se/. Cited 20 May, 2022.

[37] Reed E, Salazar M, Agah N, Behar AI, Silverman JG, Walsh-Buhi E (2019). Experiencing sexual harassment by males and associated substance use & poor mental health outcomes among adolescent girls in the US. SSM Popul Health.

[38] Mäkelä P, Raitasalo K, Wahlbeck K (2015). Mental health and alcohol use: a cross-sectional study of the Finnish general population. Eur J Pub Health.

[39] Gabrielli G, Paterno A, Strozza S (2020). Sexual behavioural differences and risk-taking differences among born-abroad and native university students in Italy. Genus.

[40] Häggström-Nordin E, Borneskog C, Eriksson M, Tydén T. Sexual behaviour and contraceptive use among Swedish high school students in two cities: comparisons between genders, study programmes, and over time. Eur J Contracept Reprod Health Care 2011;16(1):36–46. Available from: 10.3109/13625187.2010.536922. 10.3109/13625187.2010.536922 21138369

[41] Björklund A, Salvanes KG, Hanushek EA, Machin SS, Woessmann L (2011). Chapter 3 - education and family background: mechanism and policies. Handbook of the economics of education.

[42] Mmari K, Kalamar AM, Brahmbhatt H, Venables E (2016). The influence of the family on adolescent sexual experience: a comparison between Baltimore and Johannesburg. PLoS One.

[43] Coyle RM, Lampe FC, Miltz AR, Sewell J, Anderson J, Apea V (2019). Associations of depression and anxiety symptoms with sexual behaviour in women and heterosexual men attending sexual health clinics: a cross-sectional study. Sex Transm Infect.

[44] Kosunen E, Kaltiala-Heino R, Rimpelä M, Laippala P (2003). Risk-taking sexual behaviour and self-reported depression in middle adolescence-a school-based survey. Child Care Health Dev.

[45] Ramrakha S, Paul C, Bell ML, Dickson N, Moffitt TE, Caspi A (2013). The relationship between multiple sex partners and anxiety, depression, and substance dependence disorders: a cohort study. Arch Sex Behav.

[46] Ein-Dor T, Hirschberger G (2012). Sexual healing: daily diary evidence that sex relieves stress for men and women in satisfying relationships. J Soc Pers Relat.

[47] Maina BW, Orindi BO, Osindo J, Ziraba AK (2020). Depressive symptoms as predictors of sexual experiences among very young adolescent girls in slum communities in Nairobi, Kenya. Int J Adolesc Youth.

[48] Turner S, Mota N, Bolton J, Sareen J (2018). Self-medication with alcohol or drugs for mood and anxiety disorders: a narrative review of the epidemiological literature. Depress Anxiety.

[49] Garcia JR, Reiber C, Massey SG, Merriwether AM (2012). Sexual hookup culture: a review. Rev Gen Psychol.

[50] Owen J, Fincham FD (2011). Young adults’ emotional reactions after hooking up encounters. Arch Sex Behav.

[51] Scanavino MDT, Ventuneac A, Abdo CHN, Tavares H, Amaral MLS, Messina B (2018). Sexual compulsivity, anxiety, depression, and sexual risk behavior among treatment-seeking men in São Paulo, Brazil. Braz. J Psychiatry.

[52] Lin C-C, Lee Y-T, Yang H-J (2017). Risky sexual behaviors as correlates of depression and suicidal ideation among male HIV test-seekers at a voluntary counseling and testing Facility in Taiwan. Asia Pac J Public Health.

[53] Wall I, Johnsdotter S. Sex as self-injury: The appearance of a new diagnostic category in Sweden. Sexualities. 0(0):13634607221077554. 10.1177/13634607221077554.

[54] Folkhälsomyndigheten. Hivinfektion – sjukdomsstatistik. Available from: https://www.folkhalsomyndigheten.se/folkhalsorapportering-statistik/statistik-a-o/sjukdomsstatistik/hivinfektion/ . Accessed 10 June 2022.

[55] Smittskyddsinstitutet. HIV i Sverige - kunskaper, attityder och beteenden hos allmänheten 1987–2011. Solna; 2013.

[56] Kalichman SC, Weinhardt L (2001). Negative affect and sexual risk behavior: comment on Crepaz and Marks (2001). Health Psychol.

[57] Otten D, Tibubos AN, Schomerus G, Brähler E, Binder H, Kruse J, et al. Similarities and differences of mental health in women and men: a systematic review of findings in three large German cohorts. Front Public Health. 2021;(9):553071. 10.3389/fpubh.2021.553071. 10.3389/fpubh.2021.553071 PMC7892592 33614574

[58] Abate KH (2013). Gender disparity in prevalence of depression among patient population: a systematic review. Ethiop J Health Sci.

[59] Fridlund VSK, Nordvik MK (2014). Condom use: the discrepancy between practice and behavioral expectations. Scand J Public Health.

[60] Folkhälsomyndigheten. HIV idag - därför pratar vi om HIV .Available from: http://www.hividag.se/darfor-pratar-vi-om-hiv/. Cited 10 April, 2022].

[61] Folkhälsomyndigheten. Mental health. Available at: https://www.folkhalsomyndigheten.se/the-public-health-agency-of-sweden/living-conditions-and-lifestyle/mental-health/. Accessed May 10, 2022.

[62] Richert T, Anderberg M, Dahlberg M (2020). Mental health problems among young people in substance abuse treatment in Sweden. Subst Abuse Treat Prev Policy.

[63] Wu, M.-J., K. Zhao, and F. Fils-Aime, Response rates of online surveys in published research: a meta-analysis*.* Comput Hum Behav Rep, 2022. 7: 100206. 10.1016/j.chbr.2022.100206

[64] Sammut R, Griscti O, Norman IJ (2021). Strategies to improve response rates to web surveys: a literature review. Int J Nurs Stud.

[65] Ouellette JA, Wood W (1998). Habit and intention in everyday life: the multiple processes by which past behavior predicts future behavior. Psychol Bull.

[66] Galea S, Tracy M (2007). Participation rates in epidemiologic studies. Ann Epidemiol.

